# High-Sensitive Microwave Humidity Sensor Using Polyvinyl Alcohol/Carboxymethyl Cellulose (PVA/CMC) Composites

**DOI:** 10.3390/s26041099

**Published:** 2026-02-08

**Authors:** Junho Yeo, Younghwan Kwon

**Affiliations:** 1 Department of Artificial Intelligence, Daegu University, 201 Daegudae-ro, Gyeongsan-si 38453, Republic of Korea; 2 Department of Energy System Engineering, Daegu University, 201 Daegudae-ro, Gyeongsan-si 38453, Republic of Korea; y_kwon@daegu.ac.kr

**Keywords:** Carboxymethyl cellulose (CMC), Polyvinyl alcohol (PVA), PVA/CMC composites, weight percentages, capacitive microwave sensor

## Abstract

This study investigates the humidity sensing characteristics of microwave sensors coated with polyvinyl alcohol/carboxymethyl cellulose (PVA/CMC) composites with different weight percentages. The microwave sensor has a band-stop filter characteristic and consists of a microstrip transmission line with an interdigital capacitor-defected ground structure (IDC-DGS). To evaluate performance, PVA/CMC composites were prepared in 100/0 (pure PVA), 90/10, 80/20, 60/40, and 0/100 (pure CMC) weight percentages. The humidity sensing capability of the IDC-DGS-based microwave sensors coated with the PVA/CMC composites with different weight percentages was compared by measuring the variations in the resonant frequency and magnitude level of the transmission coefficient. The relative humidity (RH) was changed from 40% to 90% with increments of 10% at a temperature around 25 °C. The experimental results demonstrate that the humidity sensing capability of the microwave sensor in terms of the variations in the resonant frequency and magnitude level of the transmission coefficient increased as the weight percentage of CMC content increased. Pure CMC shows enhanced humidity sensing performance compared to gelatin and PVA in terms of the percent relative frequency shift and effective relative permittivity.

## 1. Introduction

Humidity is one of the most widely measured physical quantities along with temperature across diverse sectors [1]. In recent years, accurate measurement and control of humidity has become increasingly important because it affects people and the surrounding environment across various areas, such as human health, industrial production, and environmental management [2]. Relative humidity (RH) is the percentage ratio of the current amount of water vapor in the air to the maximum (saturated) possible water vaper at the same given temperature [3], and it is most commonly used for humidity measurement because of simple sensing structure and low cost. While various humidity transduction methods exist—including electrical, optical, surface acoustic waves, piezoresistive, and magnetoelastic [4]—resistance, capacitance, or impedance methods using the electrical characteristics of humidity sensing materials have been widely used because of their advantages, such as low cost, ease of fabrication, and sensitivity across a broad range of humidity levels [5].

The detection of the RH fundamentally relies on the physical and chemical interactions between sensing materials and water molecules. Consequently, materials characterized by porous architectures and high surface-to-volume ratios are highly sought after to maximize sensitivity [6]. These humidity-sensitive materials are generally classified into five major categories: ceramics, carbon-based nano-materials, two-dimensional (2D) materials, polymers, and their respective composites [7]. Semiconducting metal oxides, perovskites, and spinel-type oxides represent the conventional ceramic approach [8]. More recently, carbon-based nano-materials—ranging from carbon black (CB), carbon dots (CDs), carbon fibers (CFs), carbon nano-coils (CNCs), carbon nano-tubes (CNTs) to nitrogen-doped carbon oxide quantum dots (NCQDs)—have demonstrated significant potential for the humidity sensing materials [9,10,11,12,13,14,15]. Furthermore, 2D materials, including graphene, graphene oxide (GO), MXene, transition metal dichalcogenides (TMDC), black phosphorus, and titanium disillicide (TiSi_2_), has introduced ultra-thin sensing platforms with good sensitivity [16,17,18,19,20,21].

Resistive polymer humidity sensors measure the RH by detecting changes in the electrical resistance or conductivity [2,22]. Polyelectrolytes, such as quaternary ammonium salt, sulfonate salt, and phosphonium salt, utilize an ionic conduction through ionic salts to detect the RH [3], whereas conducting polymers—including polyaniline (PANI), polypyrrole (PPy), poly(4-vinylpyridine) (PVP), polythiophene (PTh), poly(3,4-ethylenedioxythiophene) (PEDOT), and poly(3,4-ethylenedioxythiophene)–poly(styrene-sulfonate) (PEDOT-PSS)—use electronic conduction to modulate resistance in response to moisture levels [23,24,25,26,27,28].

Capacitive polymer humidity sensors measure the RH by detecting changes in the electrical capacitance through the variations in relative permittivity of polymers [7]. To date, various synthetic insulating polymers, such as polyimide (PI), poly(methyl methacrylate) (PMMA), poly(2-hydroxyethyl methacrylate) (PHEMA), poly(vinyl alcohol) (PVA), and polyethyleneimine (PEI), have been used [29,30,31,32,33]. Recently, biopolymers obtained from living organisms, such as alginate, agarose, chitosan, cellulose acetate butyrate (CAB), cellulose nanofibers (CNF), gelatin, konjac glucomannan (KGM), psyllium, and starch, have been extensively investigated for capacitive polymer humidity sensors offering bio-compatibility with good sensitivity [34,35,36,37,38,39,40,41,42].

Recently, composite materials incorporating diverse material classes have been extensively investigated for humidity sensors to overcome the limits of single materials and achieve enhanced sensing properties [7]. In particular, polymer-based composite materials, such as blending of different polymers, polymers with metal oxides, polymers with carbon-based nano-materials, and polymers with 2D materials, offer a versatile platform through various blending strategies [6]. Examples of polymer-polymer blends are PVA/poly acrylic acid (PAA), PANI/PVA, and polyethylene oxide (PEO)/PVA [43,44,45]. Regarding polymers with metal oxides, notable examples include zinc oxide (ZnO)/polyethylenimine (PEI), ZnO/PANI, and titanium dioxide (TiO_2_)/PPy [46,47,48]. Furthermore, researchers have investigated polymers integrated with carbon-based nano-materials, such as CB/PI, CNF/CB, PI/multi-walled CNT, and single-walled CNT/PVA [49,50,51,52]. Recently, polymer-2D material hybrids, including PI/GO, PVA/mxene, and PPy/reduced GO (rGO) have also gained significant attention [53,54,55].

PVA is a water-soluble synthetic polymer characterized by a carbon backbone combined with hydroxyl (-OH) functional groups [39]. CMC is a water-soluble derivative of cellulose with anionic carboxymethyl groups (i.e., -CH_2_COOH) bound to some of hydroxyl groups of cellulose [56], and it has been widely used as a suspending and thickening agent in the biomedical, food, pharmaceutical, and textile industries. Since CMC is not stable in a high humidity environment, a resistive humidity sensor using CMC cross-linked with epichlorohydrin (ECH) was studied [57]. Alternatively, CMC can be effectively cross-linked with PVA through the hydrogen bonding between the OH groups in PVA and the carboxymethyl groups in CMC [58]. While previous studies have characterized the differential scanning calorimetery (DSC), thermogravimetric analysis (TGA), dielectric, and electrical properties of the PVA/CMC composite with different weight percentages [59,60], the humidity sensing characteristics of the PVA/CMC composites with different weight percentages using a capacitive microwave sensor have not yet been investigated.

In this paper, the humidity sensing performance of the PVA/CMC composites across various weight percentages was evaluated. Planar microwave sensors have been widely used because of their advantages, such as their low profile, low cost, their simple structure, and their ease of fabrication [61]. To characterize these materials, we employed a high-sensitivity microwave sensor featuring a microstrip transmission line integrated with an interdigital capacitor-defected ground structure (IDC-DGS). The IDC-DGS configuration was specifically selected for its superior sensitivity to relative permittivity variations compared to alternative designs, such as single-ring, rotated single-ring, or double-ring complementary split-ring resonators [32,39,62]. The PVA/CMC composites—prepared in 100/0, 90/10, 80/20, 60/40, and 0/100 weight percentages—were coated onto the IDC-DGS region of the microwave sensors. Sensing performance was analyzed by monitoring shifts in the first resonant frequency and the corresponding magnitude of the transmission coefficient (S_21_) for the microwave sensors. Measurements were conducted under RH levels ranging from 40% to 90% (in 10% increments) at a controlled temperature of 25 °C in a commercial temperature and humidity chamber.

**Figure 1:**
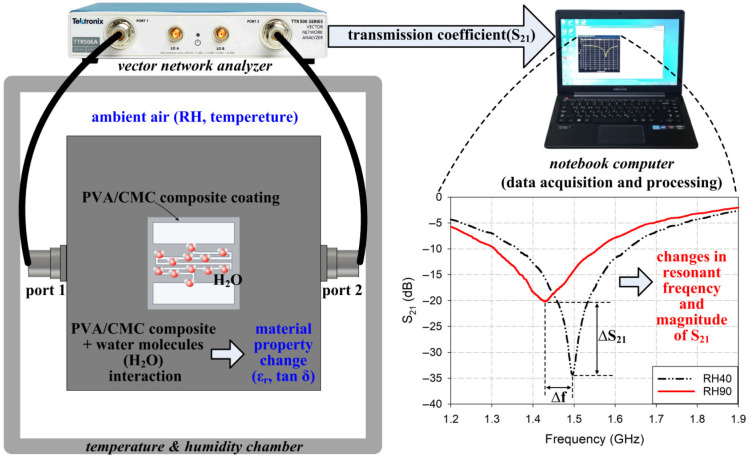
Sensing mechanism of an IDC-DGS-based microwave sensor.

Figure 1 shows the sensing mechanism of the proposed IDC-DGS-based microwave sensor coated with the PVA/CMC composite. When the coated PVA/CMC composite on the IDC-DGS-base microwave sensor is exposed to a specific RH within the environmental chamber, it undergoes a direct interaction with ambient water molecules. This interaction induces a significant modulation in the relative permittivity and loss tangent of the composite material. Specifically, as the RH increases, the higher concentration of absorbed water molecules elevates the effective relative permittivity and dielectric loss of the PVA/CMC composite, consequently leading to a downward shift in the resonant frequency of the S_21_ characteristic and a simultaneous increase in the magnitude level. These concurrent variations in the transmission characteristics serve as the fundamental mechanism for humidity detection. All electromagnetic simulations were performed using CST Studio Suite (Dassault Systèmes Co., Vélizy-Villacoublay, France) [63].

## 2. Structure and Characteristics of IDC-DGS-Based Microwave Sensor

**Figure 2:**
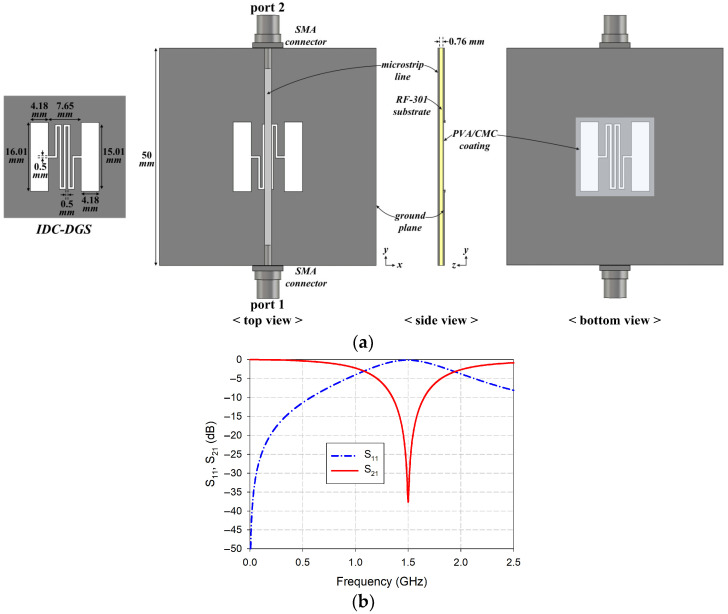
IDC-DGS-based microwave sensor. (**a**) structure; (**b**) input reflection and transmission coefficients vs. frequency.

Figure 2a shows the structure of the IDC-DGS-based microwave sensor, and its input reflection and transmission coefficients with a band-stop characteristic are shown in Figure 2b. The IDC-DGS-based microwave sensor was designed on a 50 mm × 50 mm RF-301 substrate (relative permittivity *ε*_r_ = 2.97, thickness *h* = 0.76 mm, loss tangent tan *δ* = 0.0009) to achieve the first resonant frequency (*f*_r_) of S_21_ at 1.5 GHz under unloaded (uncoated) conditions [32,39]. A 50 Ω microstrip transmission line with a width of 1.68 mm was positioned on the top side of the substrate, while the IDC-DGS was etched on the bottom ground plane. The IDC-DGS consists of a square aperture and a ridge with an IDC-shaped gap [62], with an outer dimension of 16.01 mm and gap width of 0.5 mm. Other geometric dimensions are shown in Figure 2a. To ensure complete coverage of the sensing area, the PVA/CMC composite was applied over an area of 18.05 mm in length, which is slightly larger than that of the IDC-DGS, with a measured thickness of 0.02 mm [32,39].

**Figure 3:**
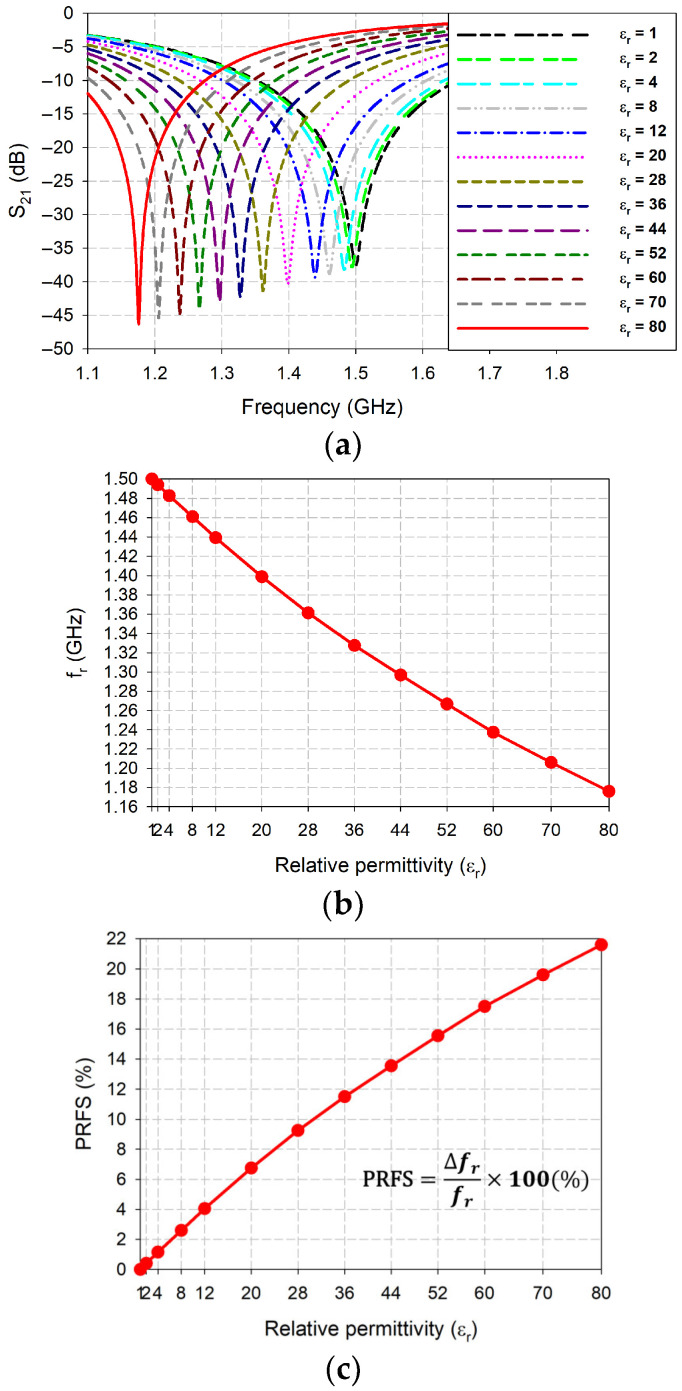
Characteristics of the IDC-DGS-based microwave sensor for varying relative permittivity of the coated dielectric polymer (tan *δ* = 0). (**a**) S_21_; (**b**) *f*_r_; (**c**) percent relative frequency shift (PRFS).

Figure 3 illustrates the simulated performance of the IDC-DGS-based microwave sensor, focusing on the S_21_ responses as a function of frequency, and the first resonant frequency (*f*_r_) and the percent relative frequency shift (PRFS) of the S_21_. These parameters were analyzed as a function of the relative permittivity (*ε*_r_) for the 0.02 mm thick polymer coating, which varied from 1 to 80 (assuming tan *δ* = 0). The PRFS is defined as the percentage difference between the loaded and unloaded first resonant frequencies relative to the unloaded first resonant frequency for S_21_ [32,39,64]. As shown in Figure 3b, the first resonant frequency exhibited a cubic decline from 1.5 GHz to 1.176 GHz, when the relative permittivity increased from 1 to 80. Concurrently, the PRFS followed a cubic growth trend, rising from 0% to 21.6%.

Table 1 summarizes the simulated first resonant frequencies and the corresponding PRFS values for S_21_ responses as a function of the coating’s relative permittivity, ranging from 1 to 80.

Utilizing the simulated PRFS data for various relative permittivity values listed in Table 1, a cubic regression model was established. The mathematical relationship between the relative permittivity and PRFS was derived using the regression analysis tool in SigmaPlot software (Systat Software Inc., San Jose, CA, USA) [65], as expressed in the following equation:

(1) $$\varepsilon_{r}=C_{0}+C_{1}\times PRFS+C_{2}\times {\left(PRFS\right)}^{2}+C_{3}\times {\left(PRFS\right)}^{3}$$

where $C_{0}=1.0513$, $C_{1}=2.5286$, $C_{2}=0.0368$, and $C_{3}=0.0007$.

## 3. Experiment Results and Discussion

**Figure 4:**
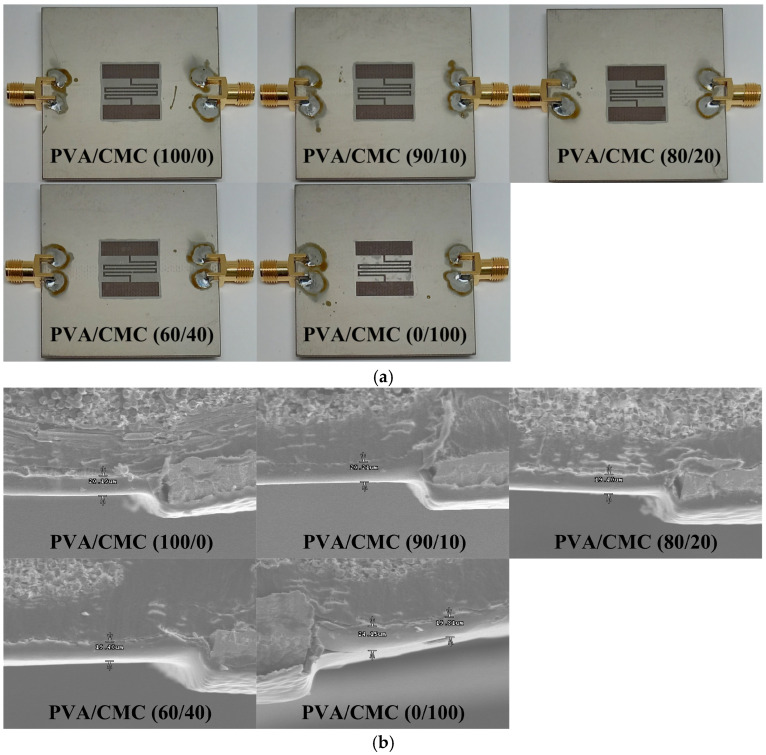
(**a**) Prototype photographs and (**b**) cross-sectional scanning electron microscopy photographs of the fabricated microwave sensors coated with PVA/CMC (100/0), PVA/CMC (90/10), PVA/CMC (80/20), PVA/CMC (60/40), and PVA/CMC (0/100).

Figure 4a displays prototypes of the fabricated IDC-DGS-based microwave sensors, coated with the PVA/CMC composites at different weight percentages (100/0, 90/10, 80/20, 60/40, and 0/100). The PVA (degree of polymerization = 1500, degree of saponification = 99 mol%) was purchased from Yakuri Pure Chemicals Co., Ltd., Kyoto, Japan [32,39]. The CMC sodium salt (CMC Na) was obtained from Duksan C&P Co. Ltd., Daejeon, Republic of Korea. The fabrication process of the sensing layers was performed in four distinct stages.

First, the PVA/CMC polymer composite solutions with a concentration of 5 wt% using different weight percentages of 100/0, 90/10, 80/20, 60/40, and 0/100 were prepared by dissolving each PVA/CMC polymer composite using deionized water as a solvent. Second, 55 mg of each PVA/CMC polymer composite solution was spread over the IDC-DGS sensing area of each fabricated microwave sensor using a precision brush. Third, the PVA/CMC polymer composite-coated microwave sensors were primarily dried using a convection oven (OF4-10P, JEIO TECH Co., Ltd., Daejeon, Republic of Korea) for about 120 min at a temperature of 60 °C. Finally, to ensure complete solvent removal, a subsequent overnight drying process was conducted in a vacuum oven (JSOV-30T, JS Research Inc., Gongju, Republic of Korea) at a temperature of 80 °C. The average thickness of the PVA/CMC polymer composite coating was measured by using a field-emission scanning electron microscopy (S-4300, Hitachi Co., Ltd., Tokyo, Japan) at a voltage of 15 kV, and it was around 0.02 mm (20 μm), as shown in Figure 4b.

**Figure 5:**
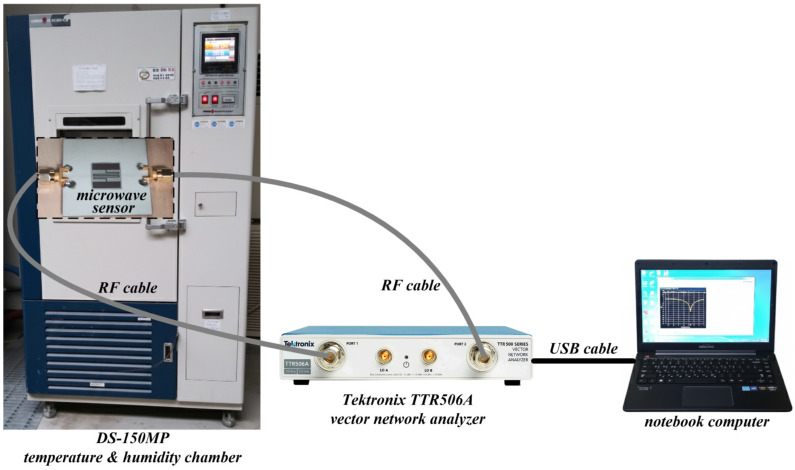
Block diagram of the humidity measurement setup.

Figure 5 shows the block diagram of the humidity measurement setup. Measurements were conducted within a calibrated commercial temperature and humidity chamber (DS-150MP, Daewon Science Inc., Bucheon, Republic of Korea). The S_21_ characteristics of the fabricated microwave sensors coated with the PVA/CMC composites at different weight percentages of 100/0, 90/10, 80/20, 60/40, and 0/100 were acquired by using the Tektronix TTR 506A (Beaverton, OR, USA) vector network analyzer.

To evaluate sensing performances, the RH of the DS-150MP temperature and humidity chamber was incrementally increased from 40% to 90% (in 10% steps) at a constant temperature of 25 °C. At each step, a 30 min stabilization period was maintained to ensure both environmental equilibrium within the chamber and complete moisture absorption by the PVA/CMC sensing layers before data were recorded on a notebook computer.

**Figure 6:**
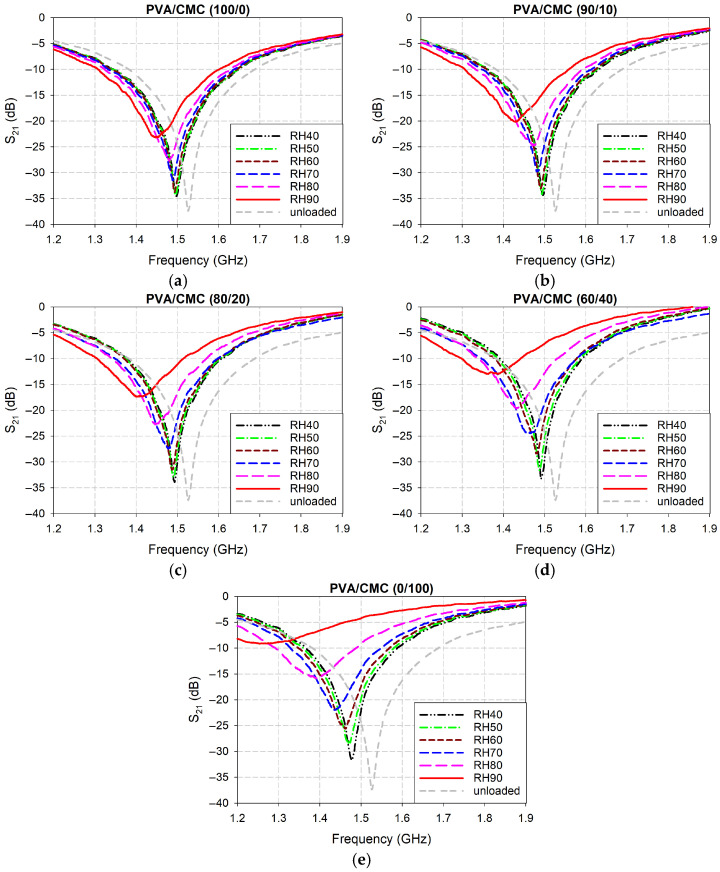
Measured S_21_ characteristics of the fabricated microwave sensors coated with the five different weight percentages of the PVA/CMC composites. (**a**) PVA/CMC (100/0), (**b**) PVA/CMC (90/10), (**c**) PVA/CMC (80/20), (**d**) PVA/CMC (60/40), and (**e**) PVA/CMC (0/100).

Figure 6 presents the measured S_21_ characteristics of the fabricated microwave sensors coated with the five different weight percentages of the PVA/CMC composites. When unloaded, the fabricated IDC-DGS-based microwave sensors exhibited the measured first resonant frequency around 1.526 GHz with a corresponding magnitude of −37.38 dB. For the pure PVA (PVA/CMC (100/0)) coating, the first resonant frequency shifted from 1.498 GHz to 1.448 GHz as the RH increased from 40% to 90%, whereas its magnitude level increased from −34.53 dB to −23.21 dB. For the PVA/CMC (90/10) coating, the first resonant frequency moved from 1.496 GHz to 1.432 GHz, whereas its magnitude level rose from −34.37 dB to −20.17 dB. For the PVA/CMC (80/20) coating, the first resonant frequency shifted from 1.493 GHz to 1.402 GHz, whereas its magnitude level increased from −33.90 dB to −17.39 dB. For the PVA/CMC (60/40) coating, the first resonant frequency decreased from 1.491 GHz to 1.363 GHz, whereas its magnitude level moved from −33.31 dB to −13.00 dB. Finally, for the pure CMC (PVA/CMC (0/100)) coating, the first resonant frequency shifted from 1.477 GHz to 1.254 GHz, whereas its magnitude level increased from −31.67 dB to −9.15 dB. It is observed that as the CMC weight percentage increased, the first resonant frequency underwent a more pronounced downward shift and a corresponding upward trend in magnitude.

Table 2, Table 3, Table 4, Table 5 and Table 6 summarize the values of the measured first resonant frequencies, PRFSs, magnitude levels, and percent relative magnitude shifts (PRMSs) of the fabricated IDC-DGS-based microwave humidity sensors coated with the five different weight percentages of the PVA/CMC composites. The PRMS represents the percentage difference between the magnitude levels at the loaded and unloaded first resonant frequencies relative to the magnitude level at the unloaded first resonant frequency [32,39].

**Figure 7:**
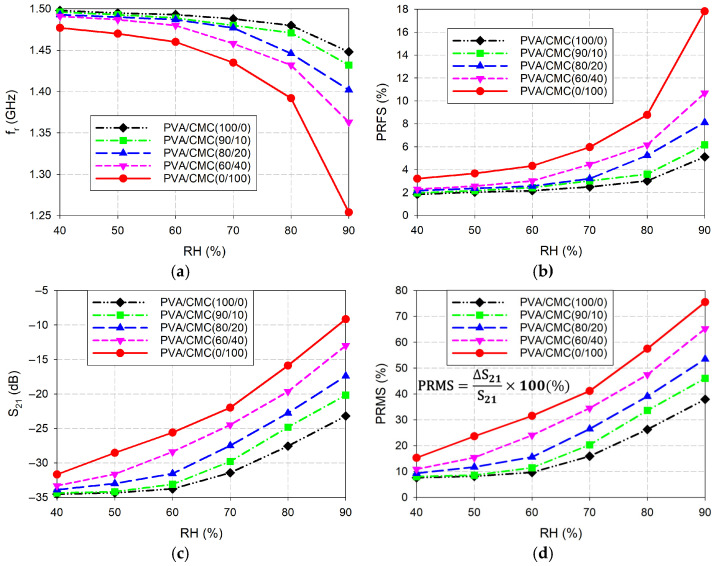
Performance comparison of the fabricated IDC-DGS-based microwave sensors coated with the five different weight percentages of the PVA/CMC composites. (**a**) *f*_r_, (**b**) PRFS, (**c**) S_21_ magnitude, and (**d**) PRMS.

The graphs of the measured first resonant frequencies, PRFSs, magnitude levels, and PRMSs of the fabricated IDC-DGS-based microwave humidity sensors coated with the five different weight percentages of the PVA/CMC composites as a function of the RH, which are obtained from Table 2, Table 3, Table 4, Table 5 and Table 6, are shown in Figure 7. For pure PVA (PVA/CMC (100/0)) coating, the PRFS increased 2.79 times as the RH increased from 40% to 90%, while the PRMS increased 4.97 times. For the PVA/CMC (90/10) coating, the PRFS and PRMS increased by factors of 3.13 and 5.73, respectively. The PVA/CMC (80/20) composite-coated IDC-DGS-based microwave sensor exhibited a 3.76-fold increase in PRFS and a 5.74-fold increase in PRMS. For the PVA/CMC (60/40) coating, the PRFS increased 4.66 times, while the PRMS increased 6.0 times. For pure CMC (PVA/CMC (0/100)) coating, the PRFS and PRMS increased by factors of 5.55 and 4.95, respectively. Analysis of the sensing parameters revealed distinct trends for PRFS and PRMS relative to the CMC concentration. The PRFS variation exhibited a monotonic increase, with the growth factor rising from 2.79 to 5.55 as the CMC weight percentage increased. In contrast, the PRMS variation followed a non-monotonic trend; it initially ascended from 4.97 to a peak of 6.0 for the PVA/CMC (60/40) composite, before declining to 4.95 for the pure CMC (PVA/CMC 0/100) coating. Both PVA and CMC are hydrophilic polymers, but they possess different types of active sites for water molecule interaction. While PVA relies only on hydroxyl (-OH) groups, CMC contains a high density of carboxymethyl groups (-CH_2_COONa) along with hydroxyl groups. As the CMC ratio increases, the concentration of carboxylate groups and sodium ions within the composite matrix rises. These groups act as high-affinity sites for water vapor adsorption through hydrogen bonding and ion-dipole interactions. A higher CMC fraction facilitates a greater volume of moisture uptake. Since water has a high relative permittivity around 80 with high loss tangent, its increased presence significantly boosts the effective permittivity and loss tangent of the composite, leading to the observed variations in the PRFS and PRMS [58,59,60]. While the present results provide valuable insights into the sensing mechanism of the PVA/CMC composite, they are based on a single experiment. Subsequent statistical verification across multiple trials is necessary to ensure the reproducibility and generalizability of the findings.

**Figure 8:**
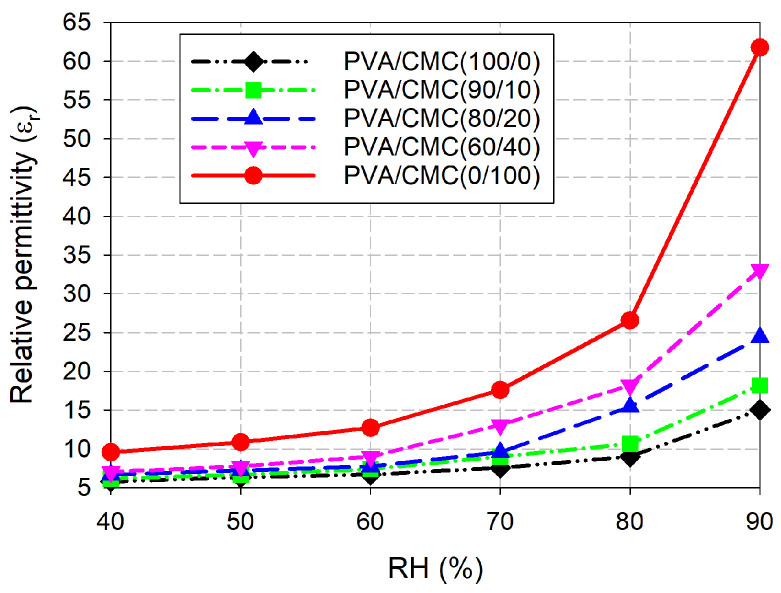
Comparison of the extracted relative permittivity for the fabricated IDC-DGS-based microwave sensors coated with the five different weight percentages of the PVA/CMC composites.

Figure 8 shows the relative permittivity of the fabricated IDC-DGS-based microwave sensors coated with the five different weight percentages of the PVA/CMC composites, extracted by applying the measured PRFS data to Equation (1). For pure PVA (PVA/CMC (100/0)) coating, the relative permittivity increased 2.58 times from 5.82 to 15.03 as the RH increased from 40% to 90%. For the PVA/CMC (90/10) coating, the relative permittivity increased by a factor of 2.95 (6.17 to 18.19). The PVA/CMC (80/20) composite-coated IDC-DGS-based microwave sensor exhibited a 3.64-fold increase (6.70 to 24.40) in the relative permittivity. For the PVA/CMC (60/40) coating, the relative permittivity increased 4.70 times from 7.05 to 33.11. The pure CMC (PVA/CMC (0/100)) coating exhibited the maximum dielectric range, shifting from 9.57 to 61.78, representing a 6.46-fold enhancement in the relative permittivity. The results demonstrate a significant enhancement in dielectric response as the CMC content increases. This systematic increase in the relative permittivity correlates directly with the enhanced humidity sensitivity observed in the PRFS.

According to [39], gelatin showed a 2.19-fold increasement from 9.47 to 20.74 in relative permittivity as the RH increased from 50% to 80%. For the same increasement in the RH, pure CMC showed a 2.44-fold increasement from 10.86 to 26.57. Therefore, the humidity sensing performance of pure CMC outperforms gelatin and PVA in terms of the PRFS and effective relative permittivity. The enhanced humidity-sensing capability of CMC might be attributed to its carboxylate groups and sodium ions, which facilitates more robust interaction with water molecules. It is hypothesized that the presence of these ionic species promotes a more significant change in dielectric perturbation upon moisture adsorption.

## 4. Conclusions

The humidity sensing properties of the PVA/CMC composites with different weight percentages were studied by using the high-sensitivity IDC-DGS-based microwave sensors with a first resonant frequency at 1.5 GHz under unloaded conditions. To establish a correlation between the dielectric properties of the coating and the sensor response, S_21_ characteristics as a function of frequency, and their first resonant frequency and PRFS for the IDC-DGS-based microwave sensor were simulated by varying the relative permittivity of a 0.02 mm thick polymer coating from 1 to 80 (with tan *δ* = 0). Utilizing a regression analysis tool, a cubic relationship was subsequently derived to express the coating’s relative permittivity as a function of the PRFS, characterized by four specific coefficients.

Prototypes of the IDC-DGS-based microwave sensors were fabricated on RF-301 substrates and functionalized with the PVA/CMC composites at various weight ratios (100/0, 90/10, 80/20, 60/40, and 0/100). The humidity-sensing performance was evaluated by monitoring the variations in the first resonant frequency and magnitude level of the S_21_ as the RH was varied from 40% to 90% with a step of 10% at a temperature of 25 °C. The results indicated that the PRFS variation exhibited a monotonic increase, rising from 2.79 to 5.55 times as the CMC content transitioned from 0% to 100%. However, the PRMS variation showed a non-monotonic trend, peaking at 6.0 times for the PVA/CMC (60/40) composite before declining to 4.95 times for the pure CMC (PVA/CMC (0/100)) sensor.

The variations in the extracted relative permittivity of the PVA/CMC composites, derived from the measured PRFS data, exhibited a trend consistent with that of the PRFS. As the CMC weight percentage increased from 0% to 100%, the growth factor of the extracted relative permittivity showed a monotonic escalation, rising from 2.58 to 6.46 times. This strong correlation validates the use of PRFS as a reliable indicator for characterizing the dielectric fluctuations of the composite layers under varying humidity. In addition, comparative analysis reveals that the humidity sensing performance of pure CMC outperforms gelatin and PVA in terms of the PRFS and effective relative permittivity.

Consequently, pure CMC can be a highly viable candidate to replace conventional PVA in humidity-sensing materials in diverse microwave applications, including chipless RFID tags, wireless sensors, and wearable electronics. Future research will focus on a comprehensive characterization of PVA/CMC composites, specifically addressing their long-term mechanical and chemical stability. Furthermore, essential sensor parameters, including repeatability, hysteresis, and response/recovery time, will be rigorously evaluated to ensure suitability for practical applications.

## Figures and Tables

**Table 1:** First resonant frequencies and PRFSs of S_21_ characteristics for the IDC-DGS -based microwave sensor for varying the relative permittivity of the coated polymer with tan *δ* = 0.

	*ε*_r_ = 1	*ε*_r_ = 2	*ε*_r_ = 4	*ε*_r_ = 8	*ε*_r_ = 12	*ε*_r_ = 20	*ε*_r_ = 28	*ε*_r_ = 36	*ε*_r_ = 44	*ε*_r_ = 52	*ε*_r_ = 60	*ε*_r_ = 70	*ε*_r_ = 80
*f*_r_ (GHz)	1.5	1.4940	1.4828	1.4610	1.4393	1.3987	1.3613	1.3275	1.2967	1.2668	1.2375	1.206	1.176
PRFS (%)	0	0.4	1.15	2.6	4.05	6.75	9.25	11.5	13.55	15.55	17.5	19.6	21.6

**Table 2:** Measured first resonant frequencies, PRFSs, magnitude levels, and PRMSs of S_21_ characteristics of the fabricated microwave sensor coated with the PVA/CMC (100/0) (pure PVA) for varying RH.

PVA/CMC (100/0)	Unloaded	RH40	RH50	RH60	RH70	RH80	RH90
*f*_r_ (GHz)	1.526	1.498	1.495	1.493	1.488	1.480	1.448
PRFS (%)	0	1.83	2.03	2.16	2.49	3.01	5.11
S_21_ (dB)	−37.38	−34.53	−34.34	−33.77	−31.44	−27.58	−23.21
PRMS (%)	0	7.63	8.14	9.66	15.89	26.22	37.92

**Table 3:** Measured first resonant frequencies, PRFSs, magnitude levels, and PRMSs of S_21_ characteristics of the fabricated microwave sensor coated with the PVA/CMC (90/10) for varying RH.

PVA/CMC (90/10)	Unloaded	RH40	RH50	RH60	RH70	RH80	RH90
*f*_r_ (GHz)	1.526	1.496	1.493	1.489	1.480	1.471	1.423
PRFS (%)	0	1.97	2.16	2.42	3.01	3.60	6.16
S_21_ (dB)	−37.38	−34.37	−34.17	−33.11	−29.79	−24.83	−20.17
PRMS (%)	0	8.04	8.58	11.43	20.29	33.58	46.05

**Table 4:** Measured first resonant frequencies, PRFSs, magnitude levels, and PRMSs of S_21_ characteristics of the fabricated microwave sensor coated with the PVA/CMC (80/20) for varying RH.

PVA/CMC (80/20)	Unloaded	RH40	RH50	RH60	RH70	RH80	RH90
*f*_r_ (GHz)	1.526	1.493	1.490	1.487	1.477	1.446	1.402
PRFS (%)	0	2.16	2.36	2.56	3.21	5.24	8.13
S_21_ (dB)	−37.38	−33.90	−33.00	−31.56	−27.50	−22.78	−17.39
PRMS (%)	0	9.31	11.72	15.57	26.43	39.07	53.48

**Table 5:** Measured first resonant frequencies, PRFSs, magnitude levels, and PRMSs of S_21_ characteristics of the fabricated microwave sensor coated with the PVA/CMC (60/40) for varying RH.

PVA/CMC (60/40)	Unloaded	RH40	RH50	RH60	RH70	RH80	RH90
*f*_r_ (GHz)	1.526	1.491	1.487	1.480	1.458	1.432	1.363
PRFS (%)	0	2.29	2.56	3.01	4.46	6.16	10.68
S_21_ (dB)	−37.38	−33.31	−31.64	−28.41	−24.50	−19.64	−13.00
PRMS (%)	0	10.88	15.36	24.00	34.46	47.45	65.23

**Table 6:** Measured first resonant frequencies, PRFSs, magnitude levels, and PRMSs of S_21_ characteristics of the fabricated microwave sensor coated with the PVA/CMC (0/100) (pure CMC) for varying RH.

PVA/CMC (0/100)	Unloaded	RH40	RH50	RH60	RH70	RH80	RH90
*f*_r_ (GHz)	1.526	1.477	1.470	1.460	1.435	1.392	1.254
PRFS (%)	0	3.21	3.67	4.33	5.96	8.78	17.82
S_21_ (dB)	−37.38	−31.67	−28.55	−25.60	−22.00	−15.89	−9.15
PRMS (%)	0	15.27	23.61	31.50	41.15	57.50	75.52

## Data Availability

Data are contained within the article.
